# Effects of deficit irrigation and kaolin application on vegetative growth and fruit traits of two early ripening apple cultivars

**DOI:** 10.1186/s40659-019-0252-5

**Published:** 2019-08-12

**Authors:** Somayeh Faghih, Zabihollah Zamani, Reza Fatahi, Abdolmajid Liaghat

**Affiliations:** 1 Department of Horticulture Science, College of Agriculture and Natural Resources, University of Tehran, Karaj, 31587 Iran; 2 Department of Irrigation and Reclamation Engineering, College of Agriculture and Natural Resources, University of Tehran, Karaj, 31587 Iran

**Keywords:** Water stress, Total phenolic content, Antioxidant activity, Water use efficiency

## Abstract

**Background:**

Drought is one of the main serious problems for agriculture production which its intensity is increasing in many parts of the world, hence, improving water use efficiency is a main goal for sustainable agriculture.

**Results:**

Growth indices including relative shoot length growth (SL), relative stem diameter increase (SD) and relative trunk cross sectional area growth (TCSA) measured at the start and end of the season decreased by reducing the irrigation level. Chlorophyll index (CI) was decreased at 70% crop evapotranspiration, however water use efficiency (WUE), leaf and fruit total phenolic content (TPC), and fruit anthocyanin content (AC) were among the traits that showed increment by water deficit stress in both cultivars. Shafi-Abadi cultivar showed to be more sensitive to the water stress than ‘Golab’. Kaolin treatment improved SL, SD and CI traits, but this increase was statistically significant only for SD at 5% level. Kaolin had no significant effect on yield and water use efficiency (WUE), however, it had negative effect on yield efficiency (YE). Kaolin treatments also significantly increased fruit and leaf TPC (*P* < 0.01) but had no effect on leaf and fruit total antioxidant activity (AA), as well as fruit anthocyanin content (AC) and soluble proteins (SP).

**Conclusions:**

Irrigation at 85% ETc showed better results than 100% and 70% ETc levels for yield attributes. It seems that the more pronounced effect of kaolin on vegetative traits but not on the fruits, might be attributed to the early ripening and harvest time of the examined cultivars.

## Background

Availability of water is one of the serious challenges for present and future of the world. Drought areas are increasing in many parts of the world, which limits agricultural production. Iran with an average rainfall of 250 mm is in the dry belt of the world and about 70% of this country is in arid and semi-arid regions [1]. In areas with drought prevalence, the use of techniques for preserving and saving water in agricultural production is critical and essential. One of the methods for water saving is deficit irrigation of the plants. Deficit irrigation (DI) reduces the vegetative growth of trees and subsequently reduces its competitiveness to reproductive growth and significantly reduces the cost of agricultural management practices [2]. Fruits in their early stages of growth (first period of cell elongation) require fewer nutrients and are less susceptible to deficit irrigation than branches, while, deficit irrigation at this time significantly reduces the growth of the branches without affecting the growth of the fruit [3]. Control of vegetative growth under deficit irrigation conditions reduced the amount of winter pruning in peaches, pears and apples [4]. Besides the economic benefits of reducing pruning costs, controlling vegetative growth can reduce the competition for photosynthesis products between fruits and vegetative organs and thus may increase the size of fruits. Lower vegetative growth also allows better light penetration inside the trees. This will help better fruit coloring [4].

Deficit irrigation is a technique to reduce the water demand, increase water use efficiency (WUE) and optimize the yield of agricultural crops. During the periods that a plant has relatively low sensitivity to the drought stress, DI with only 70–90% of water requirement can be applied with no significant reduction in yield [5]. The effect of DI on fruit yield and quality depends on the plant species, type of agronomic practices, evapotranspiration severity, soil type and soil moisture content [5]. Therefore, the benefits of DI generally can be summarized as: (1) reducing the use of irrigation water (2) improving water productivity and (3) increasing product quality [6]. Currently, DI is a common practice in many agricultural production areas of the world, especially in arid regions. It has been reported that increasing WUE under lower irrigation water applications is due to: (1) reduced water loss through evapotranspiration, (2) decreased pests and diseases damage in the root zone [7] and (3) increased number of reproductive organs and the yield by proper balance between vegetative and reproductive parts [8]. Meanwhile, prolonged reduction of the soil moisture will reduce the cell turgidity, and finally reduce the cell division and plant growth [9].

Although reports are available on the reduction of photosynthesis and growth characteristics after DI application, however, increased fruit yield of peach trees [10], apricot [11] and pomegranate [12], are reported under DI conditions. In citrus trees under DI, the WUE was similar or greater than that of the control and the quality of the fruit was improved [13].

Water deficit decreased the chlorophyll index and soluble protein content of the leaf in grape [14] but increase in yield efficiency, phenolic content, anthocyanin and antioxidant activity of apple and sugar apple have been also reported [15, 16].

The application of particle film on plants is a technique that can be used to alleviate transpiration with reducing plant temperature, leading to lower water need of plant [17]. Particle film application is usually as a completely refined white colored kaolinite powder which is used in organic agriculture, as the products treated with kaolin spray are safe for consumption after removing. Spraying aqueous suspensions of kaolin on the surface of plants results to a white kaolin layer with high porosity that remains as a protector on the surface of leaves and fruits [17, 18].

Kaolin application on apple tree maintained the structure of photosystem II, increased the net photosynthesis rate, reduced water consumption, reduced the insect damage, controlled diseases, reduced frost damage and increased the anthocyanin content of apple fruits [19–21]. Kaolin application improved the carbon dioxide assimilation rates in apple at mid-day due to enhancement of the light reflection and distribution. This resulted to reduced solar light absorption and temperature damage on the apple leaf (and fruit), meanwhile, increased the accumulation of carbon transfer to fruit and improved the quantity and quality of apple fruits [22].

Kaolin application may reduce the ROS levels and enhance the antioxidant system of plant. This was reported for example on grapevine, that reduced ROS levels, inhibited the hydroxyl radicals and increased antioxidant compounds including phenolics, flavonoids, anthocyanin, and all key metabolites [23]. Application of kaolin on olive effectively alleviated the adverse effects of environmental conditions and resulted to a significant increase in yield but did not affect the quality of olive fruit and oil [24]. Considering the growing interest in agricultural water saving techniques, the present study investigates the effects of deficit irrigation and kaolin application on some of the physiological and morphological characteristics and fruit quality of two early ripening apple cultivars.

## Materials and methods

### Experimental site and plant material

This research was carried out at the field station of the Department of Horticultural Sciences, University of Tehran at Karaj (35°48′ N, 50° 57′ E, 1293 M elevation). The apple cultivars used for this experiment consisted of ‘Golabʼ and ‘Shafi-Abadi’ grafted on seedling rootstocks. These cultivars are popular because of their early ripening and special flavor. They are the most important early commercial Iranian apple cultivars entering the market in the late spring and early summer.

The trees were at the same age (about 25 years old) cultivated in distance of 4 m × 6 m. Soil characteristics of the experiment site are presented in Table 1. The average annual temperature, and the rainfall and evaporation during the year 2017 were 16.4 °C, 169.3 mm and 1431.8 mm, respectively. Weather data obtained from the Karaj Meteorological Station, about 5 km far from the experiment site, which is considered to be representative of the study area.

**Table 1:** Physical, chemical and hydrological characteristics of the soil in the experimental site used in this study

Soil parameters	
Particle–size analysis	
Total sand (2 > ∅ > 0.02 mm)	63%
Silt (0.02 > ∅ > 0.002 mm)	18%
Clay (∅ < 0.002 mm)	19%
Organic matter	0.89%
Chemical properties	
Total nitrogen	0.75%
Available phosphorus	18.4 ppm
Exchangeable potassium	400 ppm
pH	7.75
Electrical conductivity	2.57 dS m^−1^
Physical properties	
Field capacity (by weight)	20.6%
Wilting point (by weight)	10.2%

### Irrigation and kaolin treatments

The trees were irrigated using drip irrigation system with a 3 days intervals. Seven emitters (4 L h^−1^) per plant on a loop have been installed at 1 m distance of trunk on 16 mm tubes. Three irrigation levels (I_1_, I_2_ and I_3_) were applied as follow:

(I_1_) Full irrigation, using 100% ETc during the growing season (from May to October). (I_2_) Sustained deficit irrigation at 85% of ETc and (I_3_) sustained deficit irrigation at 70% of ETc during the growing season.

Soil moisture was determined before each irrigation period and the irrigation amount was determined using Eq. (1).

1 $${\text{I}} = {\text{ETc}} = (\uptheta_{\text{fc}} - \uptheta_{\text{i}} ){\text{ DR}}$$

where: I is the total of irrigation water (mm), θ_fc_ and θ_i_ are the volumetric soil water content at field capacity and before irrigation, respectively and DR is depth of root development of the trees.

Kaolin (Sepidan WP, Kimia Green Company, Iran) was applied as water suspension at three levels of: K_1_ = 0 (control), K_2_ = 3% and K_3_ = 6% concentrations. Spraying was performed on all the canopy for 3 times (23th of May, 2 weeks and 2 months later).

### Measurement of growth and physiological parameters

The length and diameter of four branches from four directions of each tree were measured at the beginning of the water stress treatment (early May) and at fall (early October). The relative shoot length growth (SL) and relative stem diameter growth (SD) were calculated according to Bolat et al. [25]. The trunk cross sectional area (TCSA) of the trees at the height of 20 cm above the soil level were measured at the beginning of the experiment, and also at fall. The relative TCSA growth was measured according to Forey et al [10]. At the end of the stress (early October), 10 fully developed leaves from current shoots tips were collected from each tree and their chlorophyll index (CI) were measured on the two sides of the leaf by the SPAD (502 Plus Chlorophyll Meter, Minolta) and their averages were recorded.

The electrolyte leakage of leaves was measured according to Kaya et al. [26]. For this, 10 mm diameter leaf discs, were sampled from six fully developed leaves in August and October (total of 6 discs) and placed inside a falcon containing 10 ml of deionized water. The samples were then placed on a shaker (250 rpm) for 24 h and the electrical conductivity of this medium was measured using an electrical conductivity (EC) meter (initial EC). The falcons then were autoclaved at 121 °C for 15 min to kill the leaf cells. After cooling to room temperature, the EC of this solution was recorded as the secondary EC, and the electrolyte leakage percent was calculated using the equation below.

$${\text{Electrolyte leakage percent}} = \, \left( {{{{\text{EC}}_{ 1} } \mathord{\left/ {\vphantom {{{\text{EC}}_{ 1} } {{\text{EC}}_{ 2} }}} \right. \kern-0pt} {{\text{EC}}_{ 2} }}} \right) \times { 1}00$$

### Yield attributes

Fruits from each tree were harvested separately (‘Golabʼ at 73 days after full bloom (DAFB) and ‘Shafi-Abadiʼ at 97 DAFB) and weighted, as kg per tree. Yield efficiency (YE) was calculated as the fruit produced/TCSA (kg yield/cm^2^ TCSA) measured at autumn [27]. Water use efficiency was determined by the yield of tree (kg of fruit)/amount of applied water (m^3^) [28].

### Total antioxidant activity, total phenolic content, total anthocyanin and soluble proteins

Total antioxidant activity (AA) of the leaf and fruit samples were determined by 2, 2-diphenyl-1-picrylhydrazyl free radical (DPPH) assay [29] using 0.5 g fresh samples of leaf and fruit. Total phenolic content (TPC) of leaf and fruit were measured by Folin–Ciocalteu method using a plate reader (EON, Bio Tek America) at the wavelength of 725 nm [30]. For this measurement, 0.5 g of the fresh samples of leaf (collected at three times) and mature fruit were homogenized with 1.5 ml of 80% methanol and centrifuged at 15,000*g* for 15 min, 10 μl of supernatant was removed using a sampler and added into the plate well, then 75 μl of 10% Folin–Ciocalteu was added to the well, then 75 μl of 6% sodium carbonate was added to the reaction mixture. Total phenolic content was expressed as equivalent of mg gallic acid/g FW on the base of absorbance of the sample and its comparison with the standard curve.

To calculate the total anthocyanin content (AC) of fruit, the method of differences of light absorption at different pHs (pH = 1, potassium chloride buffer and pH = 4.5, sodium acetate buffer) was used [31]. For measuring AC, 0.1 g of fruit samples were crushed in 1.5 ml of 80% methanol and transferred to a 2 ml tube. The mixture was centrifuged for 15 min at 15,000*g* at 4° C. Then 23 μl of supernatant of each sample were transferred into two separate plates and the extract in the one plate was diluted with 200 μl potassium chloride buffer and the other plate diluted with sodium acetate buffer. The light absorbance of these samples were measured by a plate reader (EON, Bio Tek America) at the wavelengths of 520 nm and 700 nm. Finally, total anthocyanin of each fruit sample calculated based on the following equation.

$${\text{A}} = {\text{ A }}\left( { 5 20 \, {-}{ 7}00} \right){\text{ pH}}_{ 4. 5} {-}{\text{ A }}\left( { 5 20 \, {-}{ 7}00} \right){\text{ pH}}_{ 1}$$

$${\text{Total anthocyanin content }}\left( {{\text{mg}}/{\text{l}}} \right) \, = {\text{ A}} \times {\text{MW}} \times {\text{DF}} \times 1000/\varepsilon$$

where A: absorbance; MW: molecular weight of the cyanidin 3 glucoside (449.2); DF: dilution factor (10), ε: molar absorptivity of cyanidin 3 glucoside (26,900).

Measurement of soluble proteins (SP) was conducted using the method described by Bradford [32]. Amount of 0.1 g of grinded fresh fruit samples were transferred to the tubes, then 1 ml of phosphate buffer (50 mM, pH = 7.8) added. After 2 min of vortex, the samples were centrifuged for 15 min at 4 °C and 13,000*g*. Finally, 10 μl of extract was added to 200 μl of Bradford solution in the plate and after 20 min, the absorbance at 595 nm was read with plate reader.

Measuring the leaves total antioxidant activity (AA) and total phenolic content (TPC) were at June (T_1_), August (T_2_) and October (T_3_), and the fruit characteristics measurements were at harvest time.

### Statistical analysis

This experiment was conducted in frame of split factorial based on randomized complete block design with three replications per treatment. The main plot was deficit irrigation levels and sub-plot was kaolin concentrations. SAS statistical system software (ver. 9.4) was used to perform analysis of variance, and means were compared using Duncan’s test.

## Results

### Plant growth and physiological parameters

Monthly water consumption in different irrigation treatments during growth period measured by TDR (Time Domain Reflectometr, Mini Trase, California (USA)) instrument. Total water used during the growing season was 10.26 m^3^ for each tree at 100% ETc treatment and 8.70 and 7.16 m^3^ for treatments with 85% and 70% ETc, respectively.

Irrigation treatments had a significant effect on relative shoot length growth (SL), relative shoot diameter growth (SD) and chlorophyll index (CI) (Table 2). The highest levels of SL, SD, TCSA growths and CI were observed at irrigation level of I_1_ or control (18.80%, 13.01%, 1.79% and 44.33, respectively) and the lowest was observed at I_3_ irrigation treatment or 70% ETc (11.09%, 3.26%, 1.65% and 31.12, respectively). Thus, as expected, at 70% ETc deficit irrigation the growth parameters for shoots and chlorophyll content of leaves were decreased compared to non-stress 100% ETc. At 85% ETc deficit irrigation also the SL and SD decreased significantly (43.24% and 28.28%, respectively). The SD was significantly affected by I_3_ treatment. Although kaolin treatments increased these traits but it only affected SD growth significantly at 5% level. K_3_ on ‘Golabʼ and K_2_ on ‘Shafi-Abadiʼ increased SD growth 30.9% and 22.3% respectively, compared to control (Table 4). The ‘Golabʼ apple without kaolin had the highest TCSA growth. K_3_ treatment increased TCSA growth 19.3% relative to K_1_ in ‘Shafi Abadi’ apple (Table 4). Between the two cultivars, the higher TCSA growth was in ‘Golabʼ (1.84%) while CI was higher in ‘Shafi-Abadiʼ (43.08) (Table 2).

**Table 2:** The effect of irrigation treatments (I_1_ = 100%, I_2_ = 85% and I_3_ = 70% ETc), kaolin application (K_1_ = 0%, K_2_ = 3% and K_3_ = 6%) and their interaction, on vegetative traits of ‘Golabʼand ‘Shafi-Abadiʼ apples

Treatments	SL (%)	SD (%)	TCSA (%)	CI
Irrigation (I)	**	**	ns	**
I_1_	18.80^a^	13.01^a^	1.79^a^	44.33^a^
I_2_	10.67^b^	8.42^b^	1.75^a^	41.25^a^
I_3_	11.09^b^	3.26^c^	1.65^a^	31.12^b^
Kaolin (K)	ns	*	ns	ns
K_1_	12.10^a^	7.39^b^	1.77^a^	37.39^a^
K_2_	14.16^a^	8.91^a^	1.71^a^	39.32^a^
K_3_	14.30^a^	8.40^ab^	1.72^a^	40.00^a^
Cultivar (CV)	ns	ns	*	**
Golab	13.69^a^	8.31a	1.84a	34.72^b^
Shafi Abadi	13.36^a^	8.16a	1.63b	43.08^a^
Interactions				
I × K	ns	ns	*	ns
I × CV	ns	ns	ns	ns
K × CV	ns	*	**	ns
I × K × CV	ns	ns	ns	*

Relative shoot length growth (SL), relative shoot diameter growth (SD), trunk cross sectional area (TCSA) and chlorophyll index (CI)

Means within each column for each treatment followed by the same letters are not significantly different at *P *≤ 0.05

*ns* not significant

* and ** significant at 5% and 1% level by Duncan test

**Fig. 1:**
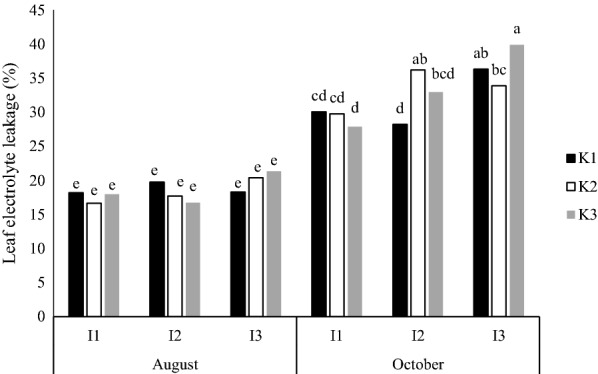
Effect of the time of the year (August and October), irrigation levels (I_1_ = 100%, I_2_ = 85% and I_3_ = 70% ETc) and kaolin application (K_1_ = 0%, K_2_ = 3% and K_3_ = 6%), on leaf electrolyte leakage percent of ‘Golabʼ and ‘Shafi-Abadiʼ apples. Columns with the same letters are not significantly different at *P *≤ 0.05 level

Interaction of irrigation and kaolin was significant for TCSA growth at 5% level. Also, interaction of kaolin and cultivar on SD was significant at 5% and for TCSA at 1% levels. The interaction of irrigation, kaolin and cultivar was significant only for CI at 5% level (Table 2). Based on the means comparison, the highest amount of SD was recorded in K_3_ treatment on ‘Golabʼ and K_2_ treatment on ‘Shafi Abadiʼ (Table 4). Golab cultivar without kaolin had the highest TCSA growth (1.99%) (Table 4). Kaolin treatments significantly improved the CI of cultivars but it was not consistent (Table 5). The highest CI (51.9) was observed at I_1_ irrigation level for K_3_Sh and the lowest CI (22.8) was in the K_1_Gb at I_3_ irrigation. K_3_ in I_3_ irrigation increased CI (44.56%) compared to K_1_ in the same irrigation (Table 5). The electrolyte leakage (EL) of leaves at August showed no significant differences, however, in October, close to leaf-fall season, the electrolyte leakage was higher than the August time (Fig. 1). This increase could be attributed to the aging of the leaves. Although at I_1_ irrigation, kaolin had no significant effect on EL, but at I_2_ irrigation, kaolin treatments increased the EL compared to control. For I_3_, the K_3_ had the highest EL of the leaves (Fig. 1).

### Yield, yield efficiency, water use efficiency and water productivity

Irrigation treatments had a significant (*P* < 0.01) effect on yield, yield efficiency (YE) and water use efficiency (WUE) (Table 3). For irrigation treatments, the highest and lowest for yield, YE and WUE were observed at of I_2_ and I_1_, respectively. I_2_ treatment compared to I_3_, increased yield, YE and WUE at 30.3%, 10.3% and 13.3% respectively. Kaolin treatment significantly reduced the YE but had no statistically significant effect on yield and WUE (Table 3). Cultivars also significantly affected yield, YE and WUE at *P* < 0.01, ‘Golabʼ showed higher values than ‘Shafi-Abadiʼ (Table 3). The analysis for interaction effect of irrigation levels by kaolin and cultivar are presented in Table 5. The highest yield for ‘Golabʼ was obtained at I_2_K_2_ treatment (72.66 kg tree^−1^) while the lowest was for I_1_K_3_ treatment (19.66 kg tree^−1^). For ‘Shafi-Abadiʼ the highest yield was at I_2_K_1_ (42.00 kg tree^−1^) while the lowest was at I_3_K_2_ (20.33 kg tree^−1^). K_3_ treatment compared to K_1_ for ‘Golabʼ and ‘Shafi-Abadiʼ in I_3_ irrigation treatment decreased yield by 7.2% and 22% respectively. Probably K_3_ treatment can exacerbate stress due to high concentration of kaolin. There was not a clear trend for yield by kaolin application and each cultivar responded differently (Table 5). I_2_ and I_3_ treatments increased YE levels by 78% and 60%, respectively (Table 3). This is due to reduced vegetative growth and reduction of competition between branches and fruits for nutritional resources. K_2_ and K_3_ treatments showed a significant reduction of 32.1% and 34.2% respectively in YE compared to control. With increasing levels of kaolin, YE decreased in both cultivars, and this decrease was higher in ‘Shafi-Abadiʼ than in the ‘Golabʼ (Table 4). The highest and the lowest WUE for ‘Golabʼ was in I_2_K_2_ (7.28 kg m^−3^) and I_1_K_3_ (1.61 kg m^−3^), respectively, while for ‘Shafi-Abadiʼ the highest was for I_2_K_1_ (4.20 kg m^−3^) and lowest for I_1_K_2_ (1.85 kg m^−3^) (Table 5).

**Table 3:** The effect of irrigation levels (I_1_ = 100%, I_2_ = 85% and I_3_ = 70% ETc) and kaolin application (K_1_ = 0%, K_2_ = 3% and K_3_ = 6%) and cultivars (Golab and Shafi Abadi) on fruit yield, yield efficiency (YE) and water use efficiency (WUE)

Treatments	Yield (kg tree^−1^)	YE (kg cm^−2^)	WUE (kg yield m^−3^ water)
Irrigation (I)	**	**	**
I_1_	26.72^c^	0.076^b^	2.22^c^
I_2_	47.16^a^	0.136^a^	4.72^a^
I_3_	32.83^b^	0.122^a^	4.09^b^
Kaolin (K)	ns	**	ns
K_1_	36.66^a^	0.143^a^	3.84^a^
K_2_	36.61^a^	0.097^b^	3.71^a^
K_3_	33.44^a^	0.094^b^	3.49^a^
Cultivar (CV)	**	**	**
Golab	43.70^a^	0.128^a^	4.54^a^
Shafi Abadi	27.44^b^	0.095^b^	2.82^b^
Interactions			
I × K	*	**	*
I × CV	**	*	**
K × CV	**	*	*
I × K × CV	*	ns	*

Means within each column for each treatment followed by the same letter are not significantly different at *P *≤ 0.05 level

*ns* not significant

* and ** showing significant effects at 5% and 1% level by Duncan test

**Table 4:** Effect of kaolin application (K_1_ = 0%, K_2_ = 3% and K_3_ = 6%) and cultivars (Golab and Shafi Abadi) on relative shoot diameter growth (SD), relative trunk cross sectional area growth (TCSA) and yield efficiency (YE)

	SD (%)		TCSA (%)		YE (kg cm^−2^)	
	‘Golabʼ	‘Shafi Abadiʼ	‘Golabʼ	‘Shafi Abadiʼ	‘Golabʼ	‘Shafi Abadiʼ
Kaolin (K)						
K_1_	7.1^b^	7.6^b^	1.99^a^	1.55^bc^	0.15^a^	0.13^a^
K_2_	8.5^ab^	9.3^a^	1.95^a^	1.48^c^	0.12^ab^	0.06^d^
K_3_	9.3^a^	7.4^b^	1.58^bc^	1.85^ab^	0.10^bc^	0.08^dc^

Means within columns for each trait followed by the same letters are not significantly different at *P *≤ 0.05 level

**Table 5:** Interaction of irrigation treatments (I_1_ = 100%, I_2_ = 85% and I_3_ = 70% ETc), kaolin application (K_1_ = 0%, K_2_ = 3% and K_3_ = 6%) and cultivars (Golab and Shafi Abadi) on chlorophyll index (CI), yield and water use efficiency (WUE)

Irrigation (I)	Kaolin (K)	CI		Yield (kg tree^−1^)		WUE (kg m^−3^)	
		Cultivar		Cultivar		Cultivar	
		Golab	Shafi Abadi	Golab	Shafi Abadi	Golab	Shafi Abadi
I_1_	K_1_	38.73^d–g^	46.90^a–c^	28.00^gh^	24.00^gh^	2.35^hj^	2.01^ij^
	K_2_	41.00^c–f^	49.43^ab^	42.00^c–f^	22.33^gh^	3.49^eh^	1.85^j^
	K_3_	37.96^e–g^	51.9^a^	19.66 ^h^	24.33^gh^	1.61^j^	2.03^ij^
I_2_	K_1_	42.43^b–e^	40.13^c–g^	50.00^b^	42.00^c–f^	5.01^b–d^	4.20^cf^
	K_2_	34.40^gf^	47.23^a–c^	72.66^a^	27.66^gh^	7.28^a^	2.77^gj^
	K_3_	37.23^e–g^	46.06^a–d^	57.66^b^	33.00^e–g^	5.77^b^	3.31^ei^
I_3_	K_1_	22.8 ^h^	33.33 ^g^	46.00^b–c^	30.00^f–h^	5.7^4b^	3.74^d–g^
	K_2_	24.96 ^h^	38.90^d–g^	34.66^d–g^	20.33 ^h^	4.33^ce^	2.51^gj^
	K_3_	32.96 ^g^	33.80^gf^	42.66^c–e^	23.33^gh^	5.32^bc^	2.91^fj^

Means within columns for each trait followed by the same letters are not significantly different at *P *≤ 0.05 level

### Chemical traits

#### Total antioxidant activity, total phenolic content, total anthocyanin and soluble proteins

Irrigation treatments did not have significant effect on total antioxidant activity (AA) of leaf and fruit and fruit total soluble proteins (SP), but had significant effect on leaf total phenolic content (TPC) and fruit TPC and anthocyanin content (AC) (Table 6). The highest levels of leaf and fruit TPC and fruit AC were observed in the I_3_ irrigation treatment. Kaolin application significantly affected leaf and fruit TPC and increased this trait (Table 6). The effect of cultivar was not significant on chemical traits. Leaf AA and TPC traits were also affected by time of the year (Table 6).

**Table 6:** Effect of the irrigation treatments (I_1_ = 100%, I_2_ = 85% and I_3_ = 70% ETc), kaolin application (K_1_ = 0%, K_2_ = 3% and K_3_ = 6%), apple cultivars (Golab and Shafi Abadi) and time of the year (June, August, October) on total antioxidant activity (AA), total phenolic content (TPC), total anthocyanin content (AC) and soluble proteins (SP) of leaves and fruits

Treatments	Leaf		Fruit			
	AA (%)	TPC (mg g^−1^ F.W)	AA (%)	TPC (mg g^−1^ F.W)	AC (mg/100 ml)	SP (mg g^−1^ F.W)
Irrigation (I)	ns	*	ns	**	**	ns
I_1_	28.23^a^	1.560^b^	36.00^a^	2.36^c^	2.24^b^	0.29^a^
I_2_	29.43^a^	1.616^ab^	38.36^a^	3.58^b^	2.53^b^	0.28^a^
I_3_	37.47^a^	1.702^a^	37.94^a^	5.95^a^	7.54^a^	0.26^a^
Kaolin (K)	ns	**	ns	**	ns	ns
K_1_	30.60^a^	1.541^b^	38.04^a^	3.35^c^	4.24^a^	0.28^a^
K_2_	32.76^a^	1.687^a^	38.08^a^	4.59^a^	3.96^a^	0.28^a^
K_3_	31.78^a^	1.650^a^	39.18^a^	3.95^b^	4.13^a^	0.27^a^
Cultivar (cv)	ns	ns	ns	ns	ns	ns
Golab	32.03^a^	1.608^a^	37.36^a^	4.14^a^	4.13^a^	0.27^a^
Shafi Abadi	31.39^a^	1.645^a^	39.50^a^	3.79^a^	4.09^a^	0.28^a^
Time	**	**	–	–	–	–
T_1_	57.61^a^	1.353^c^	–	–	–	–
T_2_	16.50^c^	1.842^a^	–	–	–	–
T_3_	21.03^b^	1.684^b^	–	–	–	–
Interactions						
I × K	ns	ns	ns	*	ns	ns
I × CV	**	ns	ns	ns	*	ns
T × I	ns	**	–	–	–	ns
K × CV	ns	ns	ns	ns	*	ns
T × K	ns	ns	–	–	–	ns
T × CV	**	ns	–	–	–	ns
I × K × CV	**	ns	ns	ns	**	ns
T × I × K	ns	ns	–	–	–	ns
T × I × CV	*	ns	–	–	–	ns
T × I × K × CV	ns	ns	–	–	–	ns

Means within each column for each treatment followed by the same letter are not significantly different at *P *≤ 0.05 level

* and ** showing significant effects at 5% and 1% level by Duncan test, ns: not significant

**Fig. 2:**
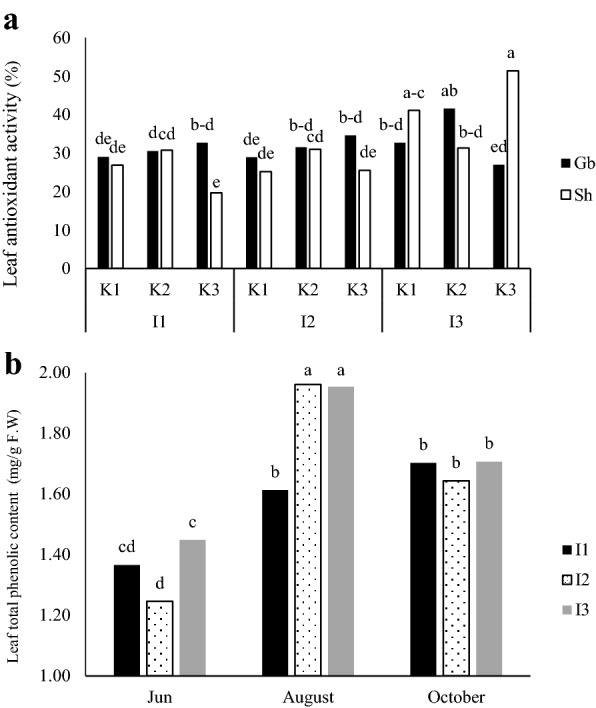
Interaction of the irrigation treatments (I_1_ = 100%, I_2_ = 85% and I_3_ = 70% ETc) and kaolin application (K_1_ = 0%, K_2_ = 3% and K_3_ = 6%), on total antioxidant activity (AA) of leaves (**a**) and interaction of the time of growth season and irrigation treatments on total phenolic content (TPC) of leaf samples (**b**) of two apple cultivars Golab and Shafi Abadi. Columns with the same letters are not significantly different at *P *≤ 0.05 level

**Fig. 3:**
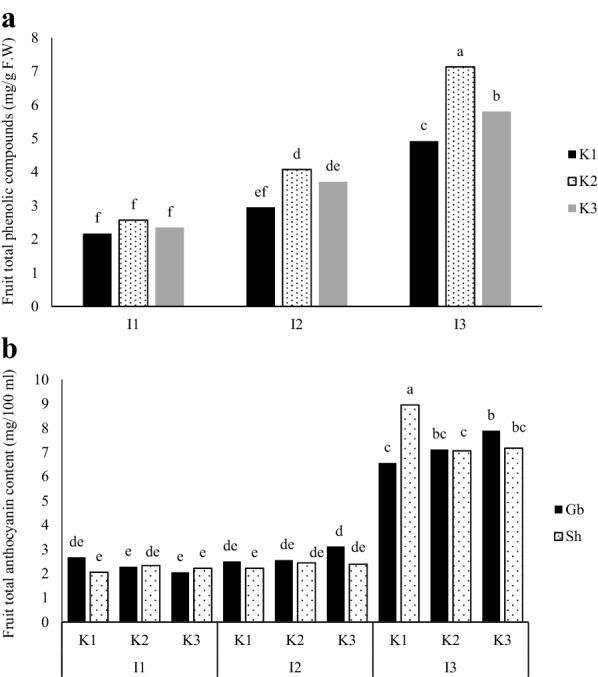
Interaction of the irrigation treatments (I_1_ = 100%, I_2_ = 85% and I_3_ = 70% ETc) and kaolin application (K_1_ = 0%, K_2_ = 3% and K_3_ = 6%) on total phenolics (**a**) and total anthocyanin (**b**) content of fruit samples of two apple cultivar (Golab and Shafi Abadi) at harvest. Columns with the same letters are not significantly different at *P *≤ 0.05 level

Based on mean comparisons for interaction affects (Fig. 2a), the highest leaf AA observed at I_3_K_3_Sh treatment (51.3%) and the lowest at I_1_K_3_Sh treatment (19.6%), but no significant difference was observed between I_1_ and I_2_ levels between kaolin and cultivar treatments (Fig. 2a). Leaf TPC was lower in early June (deficit irrigation start), then increased in August (middle of drought stress) and ultimately declined again in October (end of drought stress), coinciding with late growing season which temperature dropped and leaf abscission started. The highest levels of leaf TPC were observed in I_2_ and I_3_ treatments in August (1.96 and 1.95 mg g^−1^ F.W. respectively); about 21% higher than in control plants at the same time (Fig. 2b). Based on mean comparisons for interactions (Fig. 3a), the highest amount of fruit TPC obtained from I_3_K_3_ treatment (7.13 mg g^−1^ F.W), which was 31% higher than I_3_K_1_ and 18.6% higher than I_3_K_2_ and the lowest was in I_1_ treatment which was significantly lower compared to I_2_ and I_3_ irrigation levels (Fig. 3a). Hence, with decreasing the amount of available water in apple, the amount of phenolic content increased. Kaolin treatment at 3% increased the amount of phenolic content. The highest amount of fruit AC was recorded in 70% ETc irrigation for I_3_K_1_ on ‘Shafi-Abadiʼ compared to the other two irrigation levels (Fig. 3b). Fruit soluble proteins were not affected significantly by the treatments (data not shown).

## Discussion

Irrigation treatments had a significant effect on relative shoot length growth (SL), relative shoot diameter growth (SD) and chlorophyll index (CI) (Table 2). The SD was significantly affected by I_3_ treatment (Table 2). Bolat et al. [25] investigated the effects of different irrigation treatments including 100%, 75% and 50% FC from mid-July to the beginning of the fall period on the morphological, physiological and biochemical characteristics of the ‘Santa Mariaʼ apple scion grafted on the M9 apple and MA quince rootstocks. Deficit irrigation had a significant effect on the vegetative traits of the scion on both rootstocks. With increasing deficit irrigation, the length and diameter of the branches significantly decreased, which is similar to the present results. This is due to the higher sensitivity of vegetative growth compared to reproductive growth to deficit irrigation conditions. The increase of ABA biosynthesis in the roots and the reduction of cytokinin synthesis in the roots, branches and buds in deficit irrigation affects the vegetative growth [33]. It has also been reported that, rootstock and scion diameter of young apple trees were affected by water deficit treatments of 70% and 55% of the field capacity from July 7th until August 22nd and significantly decreased compared with the full irrigation (FC 100%) [34]. The highest levels of SL, SD, TCSA growths and CI were observed at irrigation level of I_1_ or control and the lowest was observed at I_3_ irrigation treatment or 70% ETc (Table 2). Similar to the results of present study, there are several reports about decrease in trunk growth in different fruit species including *Citrus* [13], Japanese plum [35] and apricot [11] under drought stress conditions. In line with the results of present study that 70% ETc treatment reduced the chlorophyll index, Trigo-Córdoba et al. [36] reported that drought decreased the relative chlorophyll content on two grape cultivars under rain fed and 50% ETc irrigation treatments. Similar observations were reported about other fruit species including *Citrus* rootstocks [37] and fig trees [38]. In apple trees grafted on M9 rootstock and irrigation stopped from early summer, chlorophyll index decreased significantly in deficit irrigated trees compared to control [39]. This shows that the photosynthetic pigments are very sensitive to the drought stress, and this may result in the destruction of chlorophylls. In fact, drought, high temperatures and severe sunlight usually result in lower concentrations of plant pigments (chlorophylls and carotenoids) that make a pale green color, resulting to increased light reflection from leaf surface [40]. It is suggested that glutamate, which is a primary source for production of both chlorophylls and proline, is more used for production of proline as a protectant compatible solute under stressful condition [41]. Moreover, activation of the chlorophyllase enzyme can be another reason that can cause reduction in chlorophyll content [42].

In the present study, there was no significant difference for kaolin treatment on SL and TCSA growth (Table 2). Reports about the effect of kaolin spray on plant growth are different. There are some reports indicating that kaolin treatment could increase vegetative growth [43, 44]. This is in accordance with the results of Sugar et al. [45]. Kaolin treatments of 3% and 6%, which were applied on apple trees every week from petal fall to harvest, reduced the canopy and leaf temperature but did not have a significant effect on chlorophyll content [19]. Gharaghani et al. [46] reported that the use of 3% and 6% kaolin prevented thermal and light stress in walnut trees and increased the chlorophyll content by 11.9% compared to the control trees. It is stated that the chlorophyll content in plants growing under high temperature stress might be reduced, resulting to the reduction of light absorption by plant. The results of present study confirm the positive effects of kaolin in reducing stress on apple leaves. Leaf electrolyte leakage were not significantly affected by different treatments at the August sampling, but at October it was higher in I_3_ (75% ETc) compared to I_1_ (100% ETc) (Fig. 1). Ying et al. [47] reported that electrolyte leakage in red bayberry plants significantly increased under deficit irrigation treatments compared to the control plants, which is in accordance with the results of present research. Electrolyte leakage in sensitive almond genotypes grafted on the GF677 rootstock increased by 43% at water shortage condition [48]. Severe water stress in coconut (*Cocos nucifera*) trees did not increase the electrolyte leakage, but increased under full irrigation [49]. This might be attributed to the differences in plant natures. Pear trees treated with kaolin at 3% and 6% concentrations had 10% reduced electrolyte leakage compared to control [50]. While in present study kaolin had no pronounced effect on EL of the leaves (Fig. 1).

Present results indicate that irrigation treatments at 85 and 70% ETc levels increased the yield, yield efficiency and WUE compared to control (Table 3). In accordance with present results, Alikhani-Koupaei et al. [51] reported that 70% ETc irrigation treatment significantly increased date palm cluster weight and yield, while 100% ETc treatment had the lowest cluster weight and yield. There are other reports approving yield increase under water deficit irrigation in some fruit trees including apple [52], Japanese plum [53] and pear [54]. As reported by Ebel et al. [15], RDI treatment on ‘Delicious’ apple trees reduced stem water potential, stomatal conductance and fruit growth rate, and trees treated with RDI had similar vegetative growth or less than control trees, but their yield were similar or greater than the control trees. However, the size of fruit at harvest time was influenced by the interaction of irrigation treatment and crop yield. They suggested that RDI should be ended before the size of fruit is reduced. In fruit trees, total yield depends on genetics, irrigation, nutrition, climate conditions of the year, flowering behavior and cultural practices. Agricultural management practices such as thinning (by increasing the relative number of leaves to fruit and consequently improving the development of the remaining fruits) and pruning also affect fruiting. The effect of DI and partial root-zone drying (PRD) on the yield, size and quality of ‘Fujiʼ apples in 2001–2003 was studied in the semi-arid region of Washington. PRD and DI were compared from 40 days after full bloom to before harvest. Soil water level was maintained for control plants over 80% of FC. While DI and PRD received irrigation at about 50% of control in 2001–2002 and 60% in 2003. By using DI and PRD treatments, about 45–50% water saving was achieved without any significant effect on fruit yield and size. This might be due to the climatic and soil conditions of the region. However, with the use of DI, apple fruit yield decreased in the second year compared to the control [55].

Kaolin treatment significantly reduced the YE but had no statistically significant effect on yield and WUE (Table 3). It has been reported that the use of particle film of kaolin increased the yield of pear trees by decreasing fruit abscission, which was attributed to the reduction of pests and diseases by kaolin [56]. In ‘Empireʼ apple, kaolin application of 3% and 6% concentrations improved crop production [19] which is not in accordance with the results of present research, and ‘Golabʼ and ‘Shafi-Abadiʼ yields were less affected by kaolin due to early ripening. Kaolin application after fruit set had a positive effect on olive trees production in Mediterranean regions and the increase in the yield was due to the increase in the weight and size of the fruits [57]. Kaolin significantly increased the final fruit set and fruit weight of orange trees and significantly reduced the abscission of fruit [58].

Cultivars also significantly affected yield, YE and WUE, ‘Golabʼ showed higher values than ‘Shafi-Abadiʼ (Table 3). Sun et al. [34] set the combination of two scions (‘Pink Lady’ and ‘Qinguan’)—one rootstock (*Malus hupehensis*) under two irrigation treatments of 70% and 55% of field capacity. ‘Qinguan’ apple improved WUE more than ‘Pink Lady’ under well irrigation and drought conditions. WUE in both cultivars increased significantly at 55% FC compared to 70% FC irrigation. WUE of papaya trees was improved under PRD and regulated deficit irrigation (RDI) treatments, relative to control trees [59]. Ruiz Sanchez et al. [60] suggested that probably due to the production of ABA, the vegetative growth and stomatal conductance decreased in the grapes under PRD, but WUE increased and the fruit quality also improved which is similar to the results of current research.

Contrary to this research, it has been reported that the particle film of kaolin increased WUE in ‘Ruby Redʼ grapefruit [61]. Also, Glenn et al. [19] reported that spraying of kaolin on leaves of ‘Empire’ apple caused increasing in WUE due to lower temperature and evapotranspiration of leaves and increased stomatal conductance, leaf photosynthesis and fruit weight. However, in another report, Glenn (2010) [62] reported the decreased WUE index in the ‘Empireʼ apple which might be due to the interaction of particle film with climatic and environmental conditions which is in line with the results of present research. Kaolin application reduced leaf temperature and vapor pressure deficit (VPD) and increased stomatal conductance, photosynthesis and WUE in adult grapefruit trees during hot days [61]. The application of kaolin in field conditions was beneficial when the intensity of light increased the temperature and the VPD. They concluded that in the grapefruit production regions, where high light beams and VPDs can limit the photosynthetic capacity, kaolin application, especially on young trees or small canopy trees having more leaves exposed to direct sunlight, can improve the potential of carbon absorption [61]. In the present study, kaolin increased vegetative traits, which results to more competition of branches with fruit for absorption of carbohydrates at the early of growing season and due to the early ripening fruits in present work, there were no significant effects of kaolin application on yield and WUE (Table 3).

Deficit irrigation is used as a strategy to reduce the negative effects of irrigation on the quality of apple fruit, saving water and improving water use efficiency. In present study, deficit irrigation treatments increased the level of leaf and fruit TPC and fruit AC (Table 6). In line with these results, in peach trees, RDI reduced vegetative growth, increased exposure to sunlight, reduced vitamin C and carotenoids but increased phenolic compounds such as anthocyanin and procyanidins (to reduce oxidative damage) in the fruits [63]. According to Bolat et al. [25] the content of anthocyanin and phenolics in apple trees under deficit irrigation (50% and 75% FC) on both rootstocks of M9 and MA was more than control (100% FC), but only the phenolic content had a significant difference with control plants. The increase in deficit irrigation intensity resulted to the more activity of the peroxidase and the phenolic content in both rootstocks, although the activity of catalase and anthocyanin and proline content increased with stress intensity, but this increase was not significant [25]. ‘Galaʼ apple trees were studied at Switzerland under various irrigation treatments [64]. Results showed that effects of full irrigation and RDI did not differ for the size and quality of apple fruit (soluble solids, total phenol content and vitamin C). In present study, kaolin application significantly affected leaf and fruit TPC and increased these traits (Table 6). As reported by Dinis et al. [23], spraying of 5% kaolin on grape at the onset of ripening, inhibited the hydroxyl radicals and increased antioxidant compounds such as phenolics (40%), flavonoids (24%), anthocyanin (32%), vitamin C (12%) and all key metabolites in berries relative to control. Kaolin affected the secondary metabolism and transcription of the phenylalanine ammonia lyase and chalcone synthase genes [23]. Kaolin treatment had a little effect on grape berry size after the onset of ripening, but it affected fruit compositions. As reported by Glenn et al, the use of kaolin particle film (3% and 6%) improved the color of the ‘Empireʼ apples [65, 66]. In present study, the effect of kaolin treatments were not significant on fruit anthocyanin (Table 6). This might be because the studied cultivars are not real red skin apples and the may produce pale pink on exposed surfaces specially in Shafi-Abadi cultivar. Kaolin treatments of 3% and 5.5% were applied on late ripening ‘Granny Smith’, ‘Braeburn’, ‘Fuji’, ‘Royal Gala’ and ‘Cripps Pink’ apples and the results showed that kaolin treatments reduced sunburn in all cultivars and the color of ‘Granny Smith’ and ‘Royal Gala’ cultivars improved, but there was no effect on anthocyanin and phenolic content of fruit in all cultivars compared to control. Similar to present result, DI treatments did not affect the protein content of pear jujube fruit [67].

## Conclusion

Deficit irrigation by reducing water use during a particular or entire growth period of a crop, is a strategy for reducing irrigation water use and increasing WUE, meanwhile benefiting the increase in quality attributes of the product. Reducing irrigation levels at present research decreased vegetative growth and leaf chlorophyll index of two early ripening apple cultivars, but the kaolin treatment improved these traits. Irrigation treatment of 85% ETc (I_2_) increased the yield, YE, and WUE levels compared to 100% ETc (I_1_). However, kaolin did not have a significant effect on yield and WUE. Deficit irrigation (85% and 70% ETc), increased leaf and fruit total phenolic content (TPC) and fruit anthocyanin content (AC), also kaolin sprays increased leaf and fruit TPC. According to result of present work, application of 85% ETc DI for these early ripening apple cultivars are recommended for improving the YE and WUE as well as the quality attributes of the fruit.

## Data Availability

Not applicable.

## Abbreviations

I: irrigation
K: kaolin
Gb: ‘Golabʼ
Sh: ‘Shafi-Abadiʼ
SL: shoot length
SD: stem diameter
TCSA: trunk cross sectional area
CI: chlorophyll index
YE: yield efficiency
WUE: water use efficiency
AA: antioxidant activity
TPC: total phenolic content
AC: anthocyanin content
SP: soluble proteins
PRD: partial root-zone drying
RDI: regulated deficit irrigation
ETc: crop evapotranspiration

## References

[1] Fathian F, Morid S, Kahya E (2015). Identification of trends in hydrological and climatic variables in Urmia Lake basin, Iran. Theor Appl Climatol..

[2] Chalmers D (1981). Control of peach tree growth and productivity by regulated water supply, tree density, and summer pruning. J Am Soc Hortic Sci.

[3] Zegbe-Domınguez J, Behboudian M, Lang A, Clothier B (2003). Deficit irrigation and partial rootzone drying maintain fruit dry mass and enhance fruit quality in ‘Petopride’ processing tomato (*Lycopersicon esculentum*, Mill.). Sci Hortic.

[4] Mpelasoka B, Behboudian M, Mills T (2001). Effects of deficit irrigation on fruit maturity and quality of ‘Braeburn’ apple. Sci Hortic.

[5] Ruiz-Sánchez MC, Abrisqueta I, Conejero W, Vera J, Iván F, Víctor H, Durán Z (2018). Deficit irrigation management in early-maturing peach crop, tools, strategies and challenges for woody crops. Water scarcity and sustainable agriculture in semiarid environment.

[6] Geerts S, Raes D (2009). Deficit irrigation as an on-farm strategy to maximize crop water productivity in dry areas. Agric Water Manag.

[7] Geerts S, Raes D, Garcia M, Vacher J, Mamani R, Mendoza J, Huanca R, Morales B, Miranda R, Cusicanqui J (2008). Introducing deficit irrigation to stabilize yields of quinoa (*Chenopodium quinoa* Willd). Eur J Argon.

[8] Karam F, Kabalan R, Breidi J, Rouphael Y, Oweis T (2009). Yield and water-production functions of two durum wheat cultivars grown under different irrigation and nitrogen regimes. Agric Water Manag.

[9] Hussain M, Malik M, Farooq M, Ashraf M, Cheema M (2008). Improving drought tolerance by exogenous application of glycinebetaine and salicylic acid in sunflower. Agron Crop Sci..

[10] Forey O, Metay A, Wery J (2016). Differential effect of regulated deficit irrigation on growth and photosynthesis in young peach trees intercropped with grass. Eur J Argon..

[11] Pérez-Pastor A, Ruiz-Sánchez MC, Domingo R (2014). Effects of timing and intensity of deficit irrigation on vegetative and fruit growth of apricot trees. Agric Water Manag.

[12] Intrigliolo D, Bonet L, Nortes P, Puerto H, Nicolas E, Bartual J (2013). Pomegranate trees performance under sustained and regulated deficit irrigation. Irrig Sci..

[13] Ballester C, Castel J, Intrigliolo D, Castel J (2011). Response of ‘Clementina de Nules’ citrus trees to summer deficit irrigation Yield components and fruit composition. Agric Water Manag.

[14] Meng JF, Xu TF, Wang ZZ, Fang YL, Xi ZM, Zhang ZW (2014). The ameliorative effects of exogenous melatonin on grape cuttings under water deficient stress: antioxidant metabolites, leaf anatomy, and chloroplast morphology. J Pineal Res.

[15] Ebel RC, Proebsting EL, Evans RG (1995). Deficit irrigation to control vegetative growth in apple and monitoring fruit growth to schedule irrigation. HortScience.

[16] Kowitcharoen L, Wongs-Aree C, Setha S, Komkhuntod R, Srilaong V, Kondo A (2015). Changes in abscisic acid and antioxidant activity in sugar apples under drought conditions. Sci Hortic.

[17] Ćosić M, Djurović N, Todorović M, Maletić R, Zečević B, Stričević R (2015). Effect of irrigation regime and application of kaolin on yield, quality and water use efficiency of sweet pepper. Agric Water Manag.

[18] Dinis L-T, Ferreira H, Pinto G, Bernardo S, Correia C, Moutinho-Pereira J (2016). Kaolin-based foliar reflective film protects photosystem II structure and function in grapevine leaves exposed to heat and high solar radiation. Photosynthetica..

[19] Glenn DM, Erez A, Puterka GJ, Gundrum P (2003). Particle films affect carbon assimilation and yield in ‘Empire’ apple. J Am Soc Hortic Sci.

[20] Wand SJ, Theron KI, Ackerman J, Marais SJ (2006). Harvest and post-harvest apple fruit quality following applications of kaolin particle film in South African orchards. Sci Hortic.

[21] Glenn DM, Prado E, Erez A, McFerson J, Puterka GJ (2002). A reflective, processed-kaolin particle film affects fruit temperature, radiation reflection and solar injury in apple. J Am Soc Hortic Sci.

[22] Glenn DM (2009). Particle film mechanisms of action that reduce the effect of environmental stress in ‘Empire’ apple. J Am Soc Hortic Sci.

[23] Dinis L-T, Bernardo S, Conde A, Pimentel D, Ferreira H, Félix L, Gerós H, Correia C, Moutinho-Pereira J (2016). Kaolin exogenous application boosts antioxidant capacity and phenolic content in berries and leaves of grapevine under summer stress. J Plant Physiol.

[24] Brito C, Dinis L-T, Silva E, Gonçalves A, Matos C, Rodrigues MA, Moutinho-Pereira J, Barros A, Correia C (2018). Kaolin and salicylic acid foliar application modulate yield, quality and phytochemical composition of olive pulp and oil from rainfed trees. Sci Hortic.

[25] Bolat I, Dikilitas M, Ercisli S, Ikinci A, Tonkaz A (2014). The effect of water stress on some morphological, physiological, and biochemical characteristics and bud success on apple and quince rootstocks. Sci World J.

[26] Kaya C, Kirnak H, Higgs D, Saltali K (2002). Supplementary calcium enhances plant growth and fruit yield in strawberry cultivars grown at high (NaCl) salinity. Sci Hortic.

[27] Jemrić T, Pavičić N, Blašković D, Krapac M, Pavičić D, Kniewald Z (2003). The effect of hand and chemical fruit thinning on ‘Golden Delicious cl B’ apple fruit quality. Current studies of biotechnology, food.

[28] Parvizi H, Sepaskhah AR, Ahmadi SH (2014). Effect of drip irrigation and fertilizer regimes on fruit yields and water productivity of a pomegranate (*Punica granatum* (L.) cv. Rabab) orchard. Agric Water Manag.

[29] Kim A-N, Kim H-J, Kerr WL, Choi S-G (2017). The effect of grinding at various vacuum levels on the color, phenolics, and antioxidant properties of apple. Food Chem.

[30] Singleton VL, Rossi JA (1965). Colorimetry of total phenolics with phosphomolybdic-phosphotungstic acid reagents. Am J Enol Viticult.

[31] Çam M, Hısıl Y, Durmaz G (2009). Classification of eight pomegranate juices based on antioxidant capacity measured by four methods. Food Chem.

[32] Bradford MM (1976). A rapid and sensitive method for the quantitation of microgram quantities of protein utilizing the principle of protein-dye binding. Anal Biochem.

[33] Dodd IC (2005). Root-to-shoot signalling: assessing the roles of ‘up’ in the up and down world of long-distance signalling in planta. Plant Soil.

[34] Sun X, Yan H, Kang X, Ma F (2013). Growth, gas exchange, and water-use efficiency response of two young apple cultivars to drought stress in two scion-one rootstock grafting system. Photosynthetica..

[35] Intrigliolo D, Castel J (2010). Response of plum trees to deficit irrigation under two crop levels: tree growth, yield and fruit quality. Irrig Sci..

[36] Trigo-Córdoba E, Bouzas-Cid Y, Orriols-Fernández I, Mirás-Avalos JM (2015). Effects of deficit irrigation on the performance of grapevine (*Vitis vinifera* L.) cvs. Godello and Treixadura in Ribeiro, NW Spain. Agric Water Manag.

[37] Haifeng G, Cuina F, Xinnan L (2011). Effects of drought stress on antioxidant system of leaves from different citrus rootstocks. J Agr Sci Tech..

[38] Gholami M, Rahemi M, Kholdebarin B, Rastegar S (2012). Biochemical responses in leaves of four fig cultivars subjected to water stress and recovery. Sci Hortic.

[39] Šircelj H, Tausz M, Grill D, Batič F (2005). Biochemical responses in leaves of two apple tree cultivars subjected to progressing drought. J Plant Physiol.

[40] Chai Q, Gan Y, Zhao C, Xu H-L, Waskom RM, Niu Y, Siddique KH (2016). Regulated deficit irrigation for crop production under drought stress: a review. Agron Sustain Dev.

[41] Jaleel CA, Manivannan P, Wahid A, Farooq M, Al-Juburi HJ, Somasundaram R, Panneerselvam R (2009). Drought stress in plants: a review on morphological characteristics and pigments composition. Int J Agr Biol Eng..

[42] Farooq M, Wahid A, Kobayashi N, Fujita D, Basra S (2009). Plant drought stress: effects, mechanisms and management. Agron Sustain Dev.

[43] Ou C, Du X, Shellie K, Ross C, Qian MC (2010). Volatile compounds and sensory attributes of wine from cv Merlot (*Vitis vinifera* L.) grown under differential levels of water deficit with or without a kaolin-based, foliar reflectant particle film. J Agr Food Chem..

[44] Segura-Monroy S, Uribe-Vallejo A, Ramirez-Godoy A, Restrepo-Diaz H (2015). Effect of kaolin application on growth, water use efficiency, and leaf epidermis characteristics of *Physallis peruviana* seedlings under two irrigation regimes. J Agr Sci Tech..

[45] Sugar D, Hilton RJ, VanBuskirk PD (2005). Effects of kaolin particle film and rootstock on tree performance and fruit quality in ‘Doyenne du Comice’ pear. HortScience.

[46] Gharaghani A, Javarzari AM, Vahdati K (2018). Kaolin particle film alleviates adverse effects of light and heat stresses and improves nut and kernel quality in Persian walnut. Sci Hortic.

[47] Ying Y, Yue Y, Huang X, Wang H, Mei L, Yu W, Zheng B, Wu J (2013). Salicylic acid induces physiological and biochemical changes in three red bayberry (*Myric rubra*) genotypes under water stress. Plant Growth Regul.

[48] Karimi S, Yadollahi A, Arzani K, Imani A, Aghaalikhani M (2015). Gas-exchange response of almond genotypes to water stress. Photosynthetica..

[49] Gomes FP, Oliva MA, Mielke MS, Almeida AF, Aquino LA (2010). Osmotic adjustment, proline accumulation and cell membrane stability in leaves of *Cocos nucifera* submitted to drought stress. Sci Hortic.

[50] Colavita G, Blackhall V, Valdez S (2010). Effect of kaolin particle films on the temperature and solar injury of pear fruits. Acta Hort.

[51] Alikhani-Koupaei M, Fatahi R, Zamani Z, Salimi S (2018). Effects of deficit irrigation on some physiological traits, production and fruit quality of ‘Mazafati’ date palm and the fruit wilting and dropping disorder. Agric Water Manag.

[52] Girona J, del Campo J, Bonastre N, Paris C, Mata M, Arbones A, Marsal J. Evaluation of different irrigation strategies on apple (*Malus domestica*). Physiological and productive results. In: Proc VI international symposium on irrigation of horticultural crops, Viña del Mar, Chile, 2009. p. 54.

[53] Intrigliolo D, Castel J (2005). Effects of regulated deficit irrigation on growth and yield of young Japanese plum trees. J Hortic Sci Biotech..

[54] Cheng F, Sun H, Shi H, Zhao Z, Wang Q, Zhang J (2011). Effects of regulated deficit irrigation on the vegetative and generative properties of the pear cultivar ‘Yali’. J Agric Sci Technol J Agr Sci Tech..

[55] Leib BG, Caspari HW, Redulla CA, Andrews PK, Jabro JJ (2006). Partial rootzone drying and deficit irrigation of ‘Fuji’ apples in a semi-arid climate. Irrig Sci.

[56] Puterka GJ, Glenn DM, Sekutowski DG, Unruh TR, Jones SK (2000). Progress toward liquid formulations of particle films for insect and disease control in pear. Environ Entomol.

[57] Saour G, Makee H (2003). Effects of kaolin particle film on olive fruit yield, oil content and quality. Adv Hortic Sci..

[58] Saleh M, El-Ashry S (2006). Effect of some antitranspirants on leaf mineral content, fruit set, yield and fruit quality of ‘Washington Navelʼ and ‘Succaryʼ orange trees. J Appl Sci Res..

[59] De Lima RSN, De Assis FAMM, Martins AO, De Deus BCS, Ferraz TM, Gomes MM, De Sousa EF, Glenn DM, Campostrini E (2015). Partial rootzone drying (PRD) and regulated deficit irrigation (RDI) effects on stomatal conductance, growth, photosynthetic capacity, and water-use efficiency of papaya. Sci Hortic.

[60] Ruiz Sánchez MC, Domingo Miguel R, Castel Sánchez JR (2010). Deficit irrigation in fruit trees and vines in Spain. Span J Agric Res..

[61] Jifon JL, Syvertsen JP (2003). Kaolin particle film applications can increase photosynthesis and water use efficiency of ‘Ruby Red’ grapefruit leaves. J Am Soc Hortic Sci.

[62] Glenn DM (2010). Canopy gas exchange and water use efficiency of ‘Empire’ apple in response to particle film, irrigation, and microclimatic factors. J Am Soc Hortic Sci.

[63] Buendía B, Allende A, Nicolás E, Alarcón JJ, Gil MI (2008). Effect of regulated deficit irrigation and crop load on the antioxidant compounds of peaches. J Agric Food Chem.

[64] Chenafi A, Monney P, Arrigoni E, Boudoukha A, Carlen C (2016). Influence of irrigation strategies on productivity, fruit quality and soil-plant water status of subsurface drip-irrigated apple trees. Fruits..

[65] Glenn DM, Drake S, Abbott JA, Puterka GJ, Gundrum P (2005). Season and cultivar influence the fruit quality response of apple cultivars to particle film treatments. Hort Technology..

[66] Saure MC (1990). External control of anthocyanin formation in apple. Sci Hortic.

[67] Cui N, Du T, Kang S, Li F, Zhang J, Wang M, Li Z (2008). Regulated deficit irrigation improved fruit quality and water use efficiency of pear-jujube trees. Agric Water Manag.

